# Higher Income for Male Physicians: Findings About Salary Differences Between Male and Female Iranian Physicians

**DOI:** 10.4274/balkanmedj.galenos.2018.2018.1082

**Published:** 2019-05-10

**Authors:** Enayatollah Homaie Rad, Elham Ehsani-Chimeh, Masoumeh Najafi Gharebehlagh, Fatemeh Kokabisaghi, Satar Rezaei, Maryam Yaghoubi

**Affiliations:** 1 Social Determinants of Health Research Center, Guilan University of Medical Sciences, Rasht, Iran; 2 National Institute for Health Research, Tehran University of Medical Sciences, Tehran, Iran; 3 School of Public Health, Tehran University of Medical Sciences, Tehran, Iran; 4 Erasmus School of Health Policy and Management, Erasmus University Rotterdam, Rotterdam, Netherlands; 5 Research Center for Environmental Determinants of Health, Health Institute, Kermanshah University of Medical Sciences, Kermanshah, Iran; 6 Health Management Research Center, Baqiyatallah University of Medical Sciences, Tehran, Iran

**Keywords:** Gender inequality, Iran, physicians, salary

## Abstract

**Background:**

In recent decades, the role of women in the organizations of developed and less developed countries has increased, but little is known about gender gap in salaries of Iranian physicians.

**Aims:**

To analyze the gender gap in the salary of physicians working in public health sector of Iran and its predicting factors in 2016.

**Study Design:**

Cross sectional study.

**Methods:**

Thirty thousand eight hundred and twenty four records about characteristics of study population were extracted from national human resources for health database. Nearest neighborhood matching technique was used to find adjusted differences of salary between male and female physicians. In addition, by using Oaxaca decomposition method, the reasons for the differences were found.

**Results:**

The results showed that there was a difference of 117 dollars in monthly salaries of male and female physicians in favor of men. Differences in male and female salaries could be predicted by place of work and residency, type of specialty, type of employment and marital status.

**Conclusion:**

Gender gap in physicians’ salaries exists in public health sector of Iran. The payment methods of salaries for men and women should be revised in order to remove the inequalities.

In recent decades, the role of women in organizations of developed and less developed countries has increased. Access to family planning methods and contraceptives, as well as education and health services, and support of governments have created conditions for women to enter the labor market (1). More recently, the number of female university students has increased significantly in Iran (2). In 2016, 33687 women were studying medicine, whereas the number of male medical students was 22675. However, the participation rate of women in the labor force is less than that of men in this country; it was 65.5% for men and only 12.3% for women in 2011 (3). The gender pay gap is one of the most important factors in the lower willingness of women to enter the labor market.

Studies show that the gender pay gap leads to a decrease in both productivity and efficacy. Moreover, emotional factors besides the labor market may result in lower wages for jobs held primarily by women (2,4). This issue is not limited to a region or religion or culture; almost all over the world, women face it. For example, the gender salary gap was 26.3% in Japan, 19.1% in the United States, and 15.3% in Germany in 2012 (5,6). However, in some countries, the gap is not considerable. For instance, in 2012, it was 5.9% in Norway, 6.4% in Greece, and 8.6% in Spain (5). Studies showed that gender pay gap is often smaller among medical professionals and in the public sector. However, studies in the United States showed that it was high among university physicians (7,8). Results of another study showed that in the USA, average earnings are lower for a higher percentage of female employees working in highly skilled occupations; also, this trend is happening more robustly in jobs requiring both lower education and experience (9).

In the Ministry of Health and Medical Education of Iran, there is no apparent difference defined between the salaries of male and female physicians according to the law. The only difference is the very little monthly payment to married men as heads of households (10). However, the actual situation of the gender pay gap among Iranian physicians is unknown. Because of the importance of this issue and the lack of studies on the situation of the gender salary gap in Iran’s health sector, we analyzed the gender salary gap among physicians in this country.

## MATERIALS AND METHODS

### Data

Physicians’ salary data were obtained from the Ministry of Health and Medical Education records detailing their national code, payments, work experience, level of education, job position, marital status, workplace, and work province. We extracted all general physicians’ and specialists’ information from the Ministry of Health and Medical Education’s Deputy of Human Resource Management dataset in 2016. Because the Ministry of Health and Medical Education records include only the information of physicians who are working in the public health sector, private physicians were not studied. After cleaning data and removing unrelated samples, 30824 records were entered into the study. Ethical considerations of this study were approved by the Ethics Committee of Guilan University of Medical Sciences in 2016. The validity of the data was confirmed using a random selection of 310 samples and by contacting the studied physicians. Acquiring inform consent was not applicable to this study.

### Statistical analysis

The nearest neighbor matching technique was used to reveal the differences in payments of male and female physicians in Iran. In this method, after matching variables of job position, educational degree, workplace, work experience, type of employment, specialty, province of work, and marital status, the gender pay gap could be identified. In the nearest neighbor matching estimator, the average treatment effect coefficient shows the absolute difference between payments of men and women. In addition, two multivariate regressions were estimated to show differences. The dependent variable of regressions was payments to physicians, and independent variables included all variables used in the nearest neighbor method. In addition, the Gini index was calculated for describing the distribution of salaries between males and females separately. The Gini index has a value between 0 and 1 (11,12,13), where 0 shows complete equal distribution of salaries, and 1 shows complete unequal distribution. The index was calculated using CONINDEX package in STATA software version 13.1 (StataCorp, Texas, USA). In the end, the Oaxaca decomposition technique was used to show how proportions of the differences were related to both physicians’ occupational and individual characteristics, and how proportions came from latent reasons such as a systemic gender pay gap.

Suppose that γ is the salary of general physicians, two regression models for men and women can be designed as follows:

$\gamma_{i}\;=\;\left\{\begin{array}{l} \begin{array}{c} \begin{array}{ccc} \beta^{\mathrm{males}} & X_{i} & +\;{ɛ}_{i}^{\mathrm{males}}\;\begin{array}{c} if\;\mathrm{male} \end{array} \end{array} \end{array}\\ \begin{array}{ccc} \beta^{\mathrm{famales}} & X_{i} & +\;{ɛ}_{i}^{\mathrm{famales}}\;\begin{array}{c} if\;\mathrm{famales} \end{array} \end{array} \end{array}\right.$

Where χ_i_ is the vector of dependent variables, including occupational and individual characteristics, β is the vector of coefficients of dependent variables. Then βχ_i_ can be observed by dependent variables, and ε_i_ is the vector of residuals and includes those factors that cannot be observed by dependent variables.

The gap between the payment of men and women can be determined as:

$\begin{array}{cccccccc} ∆\gamma_{i} & = & ∆x_{i}\;\beta & + & ∆\beta \;x_{i} & + & ∆\beta ∆\;x_{i} & \end{array}$

Where ∆βχ_i_ is the gap derived from coefficients that could be predicted by study variables, ∆χ_i_β is the gap related to endowments, and ∆β∆χ_i_ is the gap from interactions. For weighting the matrix of decomposition, the Cotton Method and relative sample size of the advantaged group were used (14). All statistical analyses were conducted using STATA/SE version 13.1 (StataCorp, Texas, USA).

## RESULTS

### Characteristics of the study population

The characteristics of studied physicians are presented in Table 1. The sample consisted of 30824 physicians employed by Ministry of Health and Medical Education. Of these, 16642 (53.9%) were men, and 14182 (46.1%) were women. The unadjusted mean of monthly salary was significantly higher for men [1047.42 US$ for men and 766.61 US$ for women (with a currency rate of 38140)]. A larger percentage of women were single when compared with men (31.34% vs 11.15%). The average work experience of men was 13.67 years and was significantly higher than that of women (9.66 years). The percentage of specialists (43.73%) and subspecialists (9.78%) were significantly higher among men, whereas the percentage of general physicians was higher among women (56.49%).

A larger percentage of men and women specialized in family medicine, general medicine, internal medicine, and pediatrics, whereas most gynecologists were female. Women were less likely to be academic staff than men (25.96 vs 15.20). Permanent employment was the major type of employment among men (45.30%), whereas mandatory short-term employment after graduation (mandatory employment) was the major type of employment for women (47.16%).

Gini indexes of salary distributions for men and women are presented in Table 2. The Gini index, for men, was 0.341 (±0.001), whereas it was 0.293 (±0.002) for women. Therefore, inequality in the distribution of salaries among men is higher than it is among women. The difference in the Gini index between men and women was 0.047 and was statistically significant (p<0.001).

### Nearest neighbor matching results

After adjustment for marital status, type of employment, education, specialty, job position, workplace (hospital, university, health house, headquarter, research center), province of work, and work experience, the salary difference between men and women was 116.91 (±9.02) US$, which was statistically significant (Table 3). Salary differences based on specialty types are presented in Table 3. No gender differences in the salaries of gynecologists, otolaryngologists, anesthesiologists, dermatologists, pediatricians, infectious diseases specialists, psychiatrists, neurologists, pathologists, and hematologists were found. Gender differences in the salaries of orthopedic surgeons were higher than other specialties (844.42 US$). The salaries of male emergency medicine physicians (386.85 US$) and cardiologists (366.22 US$) were much higher than those of their female counterparts.

**Figure 1:**
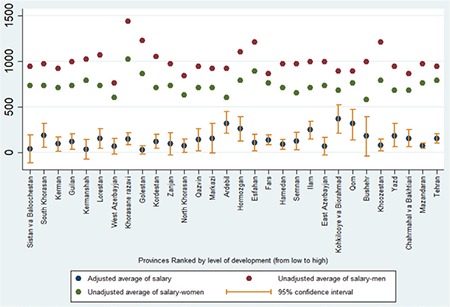
Adjusted and unadjusted gender-related differences in salary of physicians by provinces of Iran (ranked by level of development, lowest=Sistan va Baloochestan, highest=Tehran) (US$). US$: US dollars

Gender-related differences in salaries of physicians working in different provinces are presented in Figure 1. The province of Kohkiluye va boirahmad had the most considerable adjusted gender difference in salary [147.37, standard deviation: ±33.09], and Golestan had the smallest (118.76, standard deviation: 38.10). Khorasane Razavi had the highest average of unadjusted salary for both men and women (1436 vs 1012 US$), West Azerbaijan had the lowest unadjusted average of salary for men (749.86 US$), and Ardebil had the lowest for women (597.79 US$). As shown in Figure 1, the heterogeneity of adjusted salary differences between physicians of different provinces is high.

Adjusted gender-related differences in salaries for personnel with a permanent contract were more substantial than they were for individuals without a permanent contract (110.02 US$, standard deviation: 17.50). The adjusted salary difference between men and women with a short-term contract was 87.25 (±13.99) US$, for mandatory employment 64.72 (±11.64), for physicians employed for family medicine plan 9.64 (±3.51) US$, and for others 16.68 (±17.96) US$. Moreover, the adjusted salary difference between male and female specialists was 171.98 (±20.63) US$, which was higher than that of other medical professionals. Gender differences in the salaries of subspecialists were 105.21 (±33.73) US$ and for general physicians 57.35 (±3.39) US$ (Table 4).

### Oaxaca decomposition estimations

Table 5 presents the Oaxaca decomposition estimations and shows the results of decomposing gender-related differences in physicians’ salaries in Iran. According to this study, there was a 30% gender pay gap among physicians in this country. If the women studied had the same work-related characteristics as men, 21.21% of the gap could be removed. Both the type of employment (12.89%) and workplace (4.12%) had the greatest roles in creating the gap, so if women had the same type of employment as men, more than one-third of the difference in salaries could be removed. Also, education and work experience were also reasons for generating the gap (i.e., 2.14% of salary differences was related to the level of education, and 1.66% was related to work experience). Moreover, 7.14% of salary differences were related to interaction and efficiency and other factors (e.g., systematic gap and cultural factors), which could not be detected by the predictors of the study.

## DISCUSSION

In the present study, diversity in salaries of physicians employed by the Ministry of Health and Medical Education of Iran was analyzed. We used monthly payments of physicians in 2016 and collected the data from the human resource management database of the Ministry of Health and Medical Education. After adjustment of several factors including marital status, work experience, type of workplace, province of work, types of employment and education degree, we discovered there was a 116.91 US$ salary difference between male and female physicians. Average salaries of women were 30% lower when compared with those of men. Moreover, diversity can be seen in different provinces, specialties, types of employment and workplace, and different educational levels. This study, for the first time to our knowledge, used a large dataset, which was collected carefully throughout the country and showed real salaries of physicians, not revealed salaries, analyzed gender-related differences in the salaries of Iranian physicians.

Gender gap pay can be seen all over the world. Ly et al. (15) in 2016 studied the effects of gender and race differences in the salaries of physicians in the United States and showed that white male physicians have higher incomes than blacks have after adjusting for other factors. In contrast, no differences were observed between white and black female physicians’ salaries. However, differences were found in both male and female physicians’ salaries. In a study by Keshavarz Hadadian and Alavian (4), the gender-related difference in the logarithm of the hourly salary in an urban population in Iran was -0, and their study showed that women earned more than men. However, their sample was not adjusted for several important variables such as educational degree, work experience, and type of work. Using the Panel Data of the Statistical Center of Iran, Googerdchian et al. (1) found that there is a gender gap in wages of men and women in Iran. Some factors including labor participation and educational level had the greatest influence on generating the gap. However, another study found that wage differences are smaller among higher-skilled groups such as physicians (16).

This study showed that despite lower salaries of women when compared with those of men, according to the results of the Gini index, salary inequality among men was higher than it was among women. This inequality might arise from more diversity of specialty, education, and workplace among men when compared with women. In addition, the patterns of gender differences among physicians were not the same in different provinces. Skilled medical workers such as physicians are less willing to work in less developed areas, unfavorable climate conditions, and some regions with specific cultures. For improving equality in access to health professionals in these regions, Ministry of Health and Medical Education has set several incentives such as higher salaries for physicians who work in such conditions. In some provinces such as South-Khorasan, Sistan va Baloochestan, and Lorestan, the number of unbearable and less developed regions is more than other provinces, whereas, in some provinces such as Kahkilooye va Boirahmad, Ardebil, and Qom, cultural factors might affect salary differences. Male physicians tend to have more stamina to work in these regions, whereas female physicians tend to work in better weather and developed parts of provinces. Therefore, salary differences between men and women are higher in these provinces. Recently, the lack of female physicians in less developed regions has become a major concern of Ministry of Health and Medical Education (17).

Gender-related differences in the salaries of some types of specialties were higher than those of others were. For example, in orthopedic surgery, emergency medicine, and cardiology, gender differences were higher. Family physicians, social medicine specialists, and pediatricians had the least gender pay gap. Gender-related differences in salaries are related to the productivity of women at work too (1). In specialties which need more physical strength or capabilities (need for instances of more night standbys and residency in hospitals and hands-on work), women’s salaries are less.

On the other hand, orthopedic surgery and emergency medicine require less physical strength. Similar results were found in a study in the United States; adjusted gender differences of salary in orthopedic surgeries were higher than in other specialties and for family medicine, GPs, and radiologists, it was lower than in others (18). In another study in 1996, after adjustment, salary differences were higher among internal medicine and emergency medicine physicians (19). However, to our knowledge, no study has tested the relationship between the difficulty of duties of different specialties and gender difference. Studies on academic specialties showed that gender difference in salary is related to productivity. The authors calculated the productivity by the number of publications and grants (8). Different studies indicated that the productivity of female physicians was lower than that of men in academic positions (7,8,20,21,22). In 2000, Ness et al. (23) suggested that gender inequality is higher among well-paid specialties.

Academic physicians, who had managerial positions or worked at headquarter departments of medical universities, had bigger gender differences in salary when compared with other personnel. Also, temporary employed personnel had a larger gender difference compared with others. Several studies showed that there is a salary gap among academic physicians based on several variables such as work experience, type of specialty, and clinical practice expertise in the United States (9,15,18,21). More studies are needed to learn the reasons for the gender pay gap by place of work and type of employment. About one-quarter of the salary difference between men and women in Iran could not be observed by factors used in this study. Some of these factors include cultural factors, household characteristics, the responsibility of women for raising children, and the financial dependence of women on their husbands (2,4,16,24,25,26,27,28,29). However, because of the unavailability of data, a share of these variables in the gender pay gap cannot be observed or obtained easily.

The results of this study were transparent about gender differences in salaries of physicians in Iran. It used a large dataset and evaluated the gender gap using an appropriate study design. However, limitations of the study were inevitable. First, we were not able to test the effects of regional variables including the level of development of each province, and cultural factors on gender differences of salary. Second, we analyzed gender pay inequality for physicians working in the public sector. No data were available for physicians working in the private sector. In addition, we assumed that payment by the Ministry of Health and Medical Education was the sole income source of studied physicians, whereas some physicians worked in both the public and private sectors.

This study showed that there was a gap between the salaries of male and female physicians in Iran and this gap was bigger in orthopedic surgery, emergency medicine, and cardiology specialties. A large part of the gap could not be observed by the variables added in this study and may arise from cultural factors. Unified governance and comprehensive planning for the health workforce should be implemented to decrease the salary gap between different job categories and genders. Inequality in income is not a direct result of discrimination in pricing medical services based on gender. It is mainly rooted in socio-cultural and economic structures that restrict the opportunities and participation of women in this labor market. Social changes are needed to provide equal chances for men and women. Since the law does not discriminate based on gender in the payment of salaries, further research is needed to investigate the reasons for the actual disparities.

## Figures and Tables

**Table 1:**
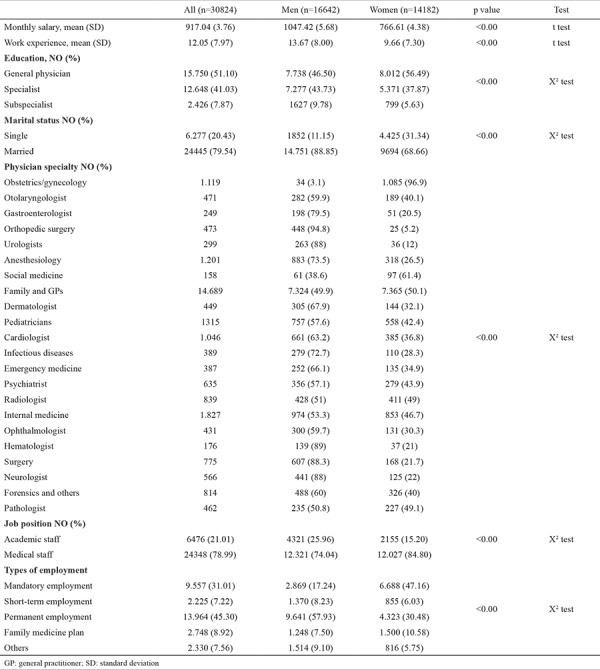
Characteristics of study population

**Table 2:**
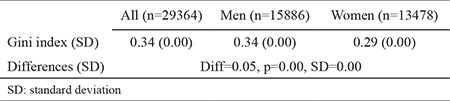
Gini index of salaries for men and women

**Table 3:**
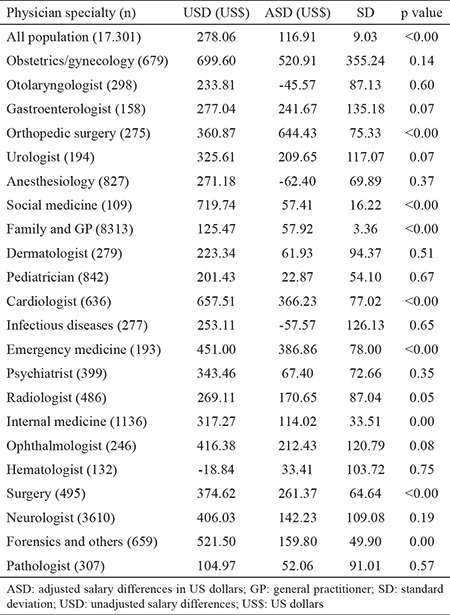
Salary differences by type of specialty before and after adjustment of effective factors

**Table 4:**
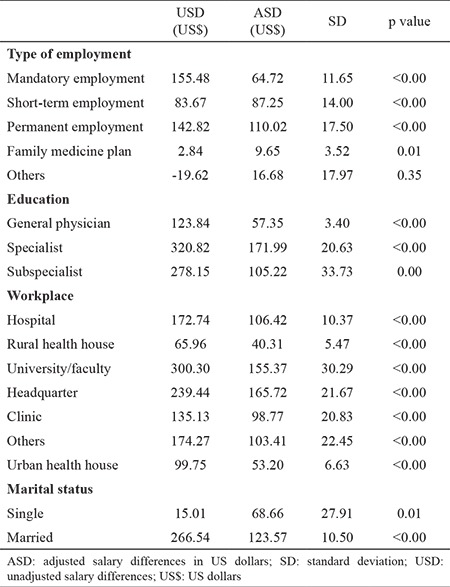
Unadjusted and adjusted salary differences between men and women by type of employment, level of education, workplace and marital status

**Table 5:**
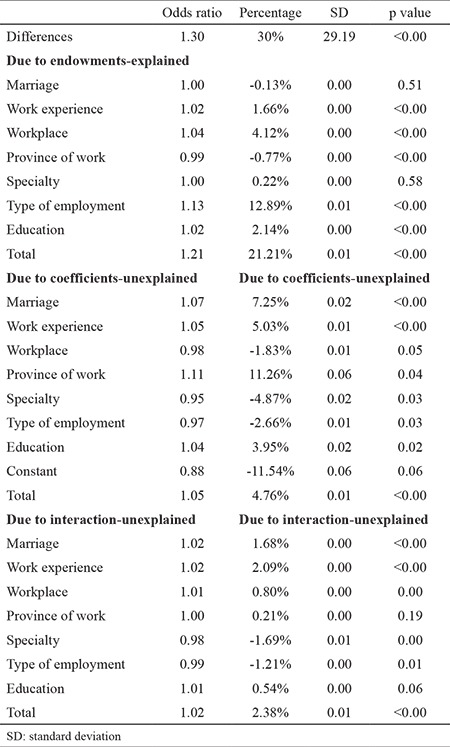
Oaxaca decomposition estimations showing gender inequality in salaries of physicians in Iran
